# Hyperosmotic Stress Initiates AMPK-Independent Autophagy and AMPK- and Autophagy-Independent Depletion of Thioredoxin 1 and Glyoxalase 2 in HT22 Nerve Cells

**DOI:** 10.1155/2019/2715810

**Published:** 2019-03-27

**Authors:** Alcir Luiz Dafre, Ariana Ern Schmitz, Pamela Maher

**Affiliations:** ^1^Biochemistry Department, Federal University of Santa Catarina, 88040-900 Florianópolis, SC, Brazil; ^2^Cellular Neurobiology Laboratory, Salk Institute for Biological Studies, CA 92037 La Jolla, USA

## Abstract

**Background:**

Hyperosmotic stress is an important pathophysiologic condition in diabetes, severe trauma, dehydration, infection, and ischemia. Furthermore, brain neuronal cells face hyperosmotic stress in ageing and Alzheimer's disease. Despite the enormous importance of knowing the homeostatic mechanisms underlying the responses of nerve cells to hyperosmotic stress, this topic has been underrepresented in the literature. Recent evidence points to autophagy induction as a hallmark of hyperosmotic stress, which has been proposed to be controlled by mTOR inhibition as a consequence of AMPK activation. We previously showed that methylglyoxal induced a decrease in the antioxidant proteins thioredoxin 1 (Trx1) and glyoxalase 2 (Glo2), which was mediated by AMPK-dependent autophagy. Thus, we hypothesized that hyperosmotic stress would have the same effect.

**Methods:**

HT22 hippocampal nerve cells were treated with NaCl (37, 75, or 150 mM), and the activation of the AMPK/mTOR pathway was investigated, as well as the levels of Trx1 and Glo2. To determine if autophagy was involved, the inhibitors bafilomycin (Baf) and chloroquine (CQ), as well as ATG5 siRNA, were used. To test for AMPK involvement, AMPK-deficient mouse embryonic fibroblasts (MEFs) were used.

**Results:**

Hyperosmotic stress induced a clear increase in autophagy, which was demonstrated by a decrease in p62 and an increase in LC3 lipidation. AMPK phosphorylation, linked to a decrease in mTOR and S6 ribosomal protein phosphorylation, was also observed. Deletion of AMPK in MEFs did not prevent autophagy induction by hyperosmotic stress, as detected by decreased p62 and increased LC3 II, or mTOR inhibition, inferred by decreased phosphorylation of P70 S6 kinase and S6 ribosomal protein. These data indicating that AMPK was not involved in autophagy activation by hyperosmotic stress were supported by a decrease in p^S555^-ULK1, an AMPK phosphorylation site. Trx1 and Glo2 levels were decreased at 6 and 18 h after treatment with 150 mM NaCl. However, this decrease in Trx1 and Glo2 in HT22 cells was not prevented by autophagy inhibition by Baf, CQ, or ATG5 siRNA. AMPK-deficient MEFs under hyperosmotic stress presented the same Trx1 and Glo2 decrease as wild-type cells.

**Conclusion:**

Hyperosmotic stress induced AMPK activation, but this was not responsible for its effects on mTOR activity or autophagy induction. Moreover, the decrease in Trx1 and Glo2 induced by hyperosmotic stress was independent of both autophagy and AMPK activation.

## 1. Introduction

Several cell types can experience hyperosmolarity such as chondrocytes during periods of prolonged mechanical loading, lymphoid tissues are constitutively hyperosmolar, and endothelial cells are constantly exposed to alterations in blood osmolarity. Accumulation of inorganic ions, protein aggregation and malfunction, molecular crowding, and DNA damage can be induced under hyperosmotic stress. To face stress conditions, cells present an adaptive response, including the ability to regulate and maintain homeostatic levels of solutes in either intracellular or extracellular microenvironments, which is vital to maintaining cellular health. Mechanisms of hyperosmotic control include remodeling the repertoire of solute transporters, as well as increasing organic osmolytes and the expression of heat shock proteins, along with a number of other cellular responses [1, 2].

Brain neurons have to cope with osmotic disturbances in blood plasma, such as plasma osmolarity associated with hypernatremia, dehydration, and diabetes. Furthermore, brain osmolarity fluctuates during the progression of traumatic brain injury, infection, and ischemia [2–4]. Reduced thirst and lower water intake are characteristics of ageing, which result in dehydration in both elderly humans and animal models of ageing [4]. Alzheimer's disease patients present an impaired response to fluid restriction, even in a mild overnight fluid restriction paradigm [5]. Increased tau phosphorylation and production of beta amyloid precursor proteins were also found in neuronal cells exposed to hyperosmotic stress, leading to altered cleavage of amyloid precursor protein, increasing beta amyloid peptides [6, 7]. Despite the enormous importance of understanding the homeostatic mechanisms underlying the response of nerve cells to osmotic stress, this topic has been underrepresented in the literature.

Hyperosmotic stress has been shown to induce autophagy in several organisms, including yeast [8], fish [9], and a number of mammalian cells [9–12]. Autophagy and microtubule remodeling have been proposed to play a prominent role in the osmoprotective cell response [12]. In this regard, mechanical sensors are important elements in activating the cell signaling pathways required for cell adaptation and survival [13]. Nevertheless, uncontrolled hyperosmotic stress can lead to apoptotic cell death [10].

Autophagy is a conserved mechanism that is constitutively active and is constantly degrading and recycling components of eukaryotic cells, but stress can increase autophagy flux [14]. Homeostatic regulation of autophagy is critical for maintaining normal energy homeostasis and metabolism. Several human diseases involve medical interventions focused on modulating autophagy, aiming at cellular adaptation and remodeling [15].

Only a few mechanistic studies addressing the hyperosmotic stress response have been published. Jiang and colleagues proposed a mechanism in nucleus pulposus intervertebral disc cells under hyperosmotic stress in which autophagy markers were dependent on 5′ AMP-activated protein kinase (AMPK) activation and consequent mechanistic target of rapamycin (mTOR) inhibition. Autophagy was demonstrated by increased beclin 1 levels and microtubule-associated protein 1 light chain 3 (LC3) lipidation, while p62 protein decreased over time [11]. Similarly, AMPK-dependent autophagy was proposed to occur in macrophages exposed to hyperosmotic stress [16]. However, the same finding was not seen in another study using nucleus pulposus cells [17].

Previously, we demonstrated that the thioredoxin (Trx) and glyoxalase (Glo) systems are molecular targets of methylglyoxal, an important glycating agent [18, 19]. Glo1 and Glo2 are responsible for eliminating the endogenous toxin methylglyoxal, which has been associated with neurodegenerative diseases [20]. Trx plays a pivotal role in the regulation of redox signaling, maintaining the balance of the thiol-related redox status [21]. Trx also participates in the regeneration of peroxiredoxins, a main peroxidase and redox relay system [22]. In our previous work, methylglyoxal produced a robust induction of autophagic markers, which was associated with a decrease in Trx1 and Glo2. This methylglyoxal-induced loss of Trx1 and Glo2 was dependent on autophagy controlled by AMPK [23]. Given the relationship between hyperosmotic stress and autophagy, and its proposed dependence on AMPK activation, we investigated whether hyperosmotic stress can affect Trx1 and Glo2. We further addressed the possible mechanisms controlling Trx1 and Glo2 levels by hyperosmotic stress by using autophagy inhibitors and siRNA, while the relationship with AMPK was studied in AMPK-deficient mouse embryonic fibroblast (MEFs).

## 2. Material and Methods

### 2.1. Cell Culture and Treatments

Immortalized mouse hippocampal HT22 cells were grown on tissue culture dishes in high-glucose DMEM that was supplemented with 10% FCS as previously published [18, 24]. Mouse embryonic fibroblasts (MEFs) were cultured under the same conditions. Wild-type and AMPK-deficient (AMPK-KO) MEFs were a generous gift of Dr. Reuben Shaw from the Salk Institute, La Jolla, CA. Following treatment with 0, 37, 75, or 150 mM NaCl, the medium was exchanged with fresh medium without NaCl added, and cell viability was quantified by the MTT assay [25]. Most experiments were performed up to 6 h, where cell viability was preserved (data not shown). Twenty-four hours after cells were treated with NaCl, the viability was (±SEM, *N* = 4) 37 mM = 96.2 ± 2.0%, NS; 75 mM = 89.9 ± 3.9%, NS; and 150 mM = 22.1 ± 7.6%, *p* < 0.01. For other assays, 3 × 10^5^ cells were plated in 60 mm dishes, and the cells were grown to semiconfluence. Prior to harvesting the cells, the medium was aspirated, and the cells were rinsed twice with cold PBS.

### 2.2. Transfection

For siRNA transfections, HT22 cells were plated in 60 mm dishes at 5 × 10^5^ cells/dish. For transfection, 166 pmol ATG5 siRNA (#sc-41446) or control siRNA (#sc-37007), all from Santa Cruz Biotechnology (Santa Cruz, CA) was used along with RNAiMAX (Invitrogen) according to the manufacturer's instructions.

### 2.3. Western Blot

HT22 or MEFs cells were plated at a density of 3 × 10^5^ cells per 60 mm dish. At 80% confluence, the cells were treated as indicated, rinsed twice in ice-cold, Tris-buffered saline, and harvested in sample buffer (25 mM Tris, pH 8.0, 2% SDS, 25 mM 2-mercaptoethanol, 1 mM Na_3_VO_4_).

Samples were analyzed by SDS-PAGE using 10% Criterion XT Precast Bis-Tris Gels (Bio-Rad). Proteins were transferred to polyvinylidene fluoride membranes and probed with the desired primary antibody as detailed elsewhere [18, 26]. Primary antibodies and their respective dilutions and sources were Glo2 (sc-31057, 1 : 1000, goat), p^S2448^-mTOR (sc-297, 1 : 1000, mouse) from Santa Cruz Biotechnology, p^T172^-AMPK (2535, 1 : 1000, rabbit), *α*-AMPK (2793, 1 : 1000, rabbit), LC3 (4108S, 1 : 1000, rabbit), Trx1 (2429, 1 : 3000, rabbit), p^S235/S236^-S6 (2211, 1 : 1000, rabbit), p^T389^-P70S6K (9255, 1 : 1000, rabbit), mTOR (2972, 1 : 1000, rabbit), p^S555^-ULK1 (5869, 1 : 1000, rabbit) from Cell Signaling, and p62 (sequestosome) (1 : 5000, guinea pig) from American Research Products.

Immunodetection was performed by using Super Signal West Pico Substrate (Pierce) with the appropriate secondary antibody. For each antibody, the same membrane was reprobed for actin or Ponceau S to normalize the protein load or as indicated. Horseradish peroxidase-conjugated secondary antibodies (Bio-Rad) were diluted 1/5000 in 5% skim milk in Tris-buffered saline/0.1% Tween 20 prior to use. Autoradiographs were scanned using a Bio-Rad GS800 scanner, and band density was measured using the Quantity One software.

### 2.4. Statistical Analysis

One-way ANOVA, followed by the Dunnett post hoc test, was used to compare the test groups with the control group. Statistical significance was set at *p* values < 0.05. Data are presented as mean ± SEM of at least 3 independent experiments, unless otherwise stated.

## 3. Results

### 3.1. AMPK/mTOR Signaling and Autophagy Markers

**Figure 1:**
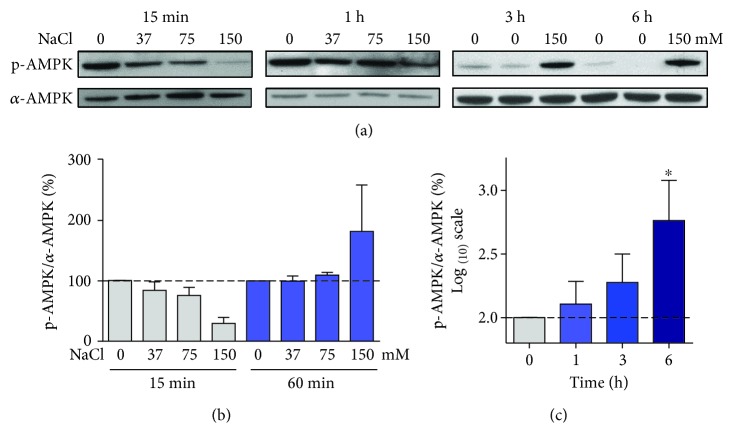
Biphasic modulation of AMPK by hyperosmotic stress in HT22 cells. Cells were treated for the indicated times and indicated NaCl concentrations. Representative blot images of the phosphorylated (p-AMPK) and total forms of (a) AMPK and their respective quantifications (b and c). (c) Data obtained with the 150 mM NaCl treatment. Values are presented as mean ± SEM, *N* = 3 − 5. Statistical significance is presented as ^∗^*p* < 0.05.

AMPK has been proposed to control autophagy [27], including in the context of cells under hyperosmotic stress [11]. Therefore, we first investigated the effects of hyperosmotic stress on AMPK phosphorylation at serine 172, an activation site [28], in the HT22 hippocampal nerve cells. Surprisingly, shortly after 150 mM NaCl treatment (15-60 min), AMPK phosphorylation was not statistically different from the control group. However, a tendency to lower levels of phosphorylation can be seen with 150 mM NaCl at 15 min (Figures 1(a) and 1(b)), while 1 h posttreatment appears to represent a transition phase where the phosphorylation showed a tendency to higher values, but without statistical significance. After 150 mM NaCl treatment, an increase in AMPK phosphorylation was observed after 3 h and at statistically significant levels at 6 h (Figures 1(a) and 1(c)).

**Figure 2:**
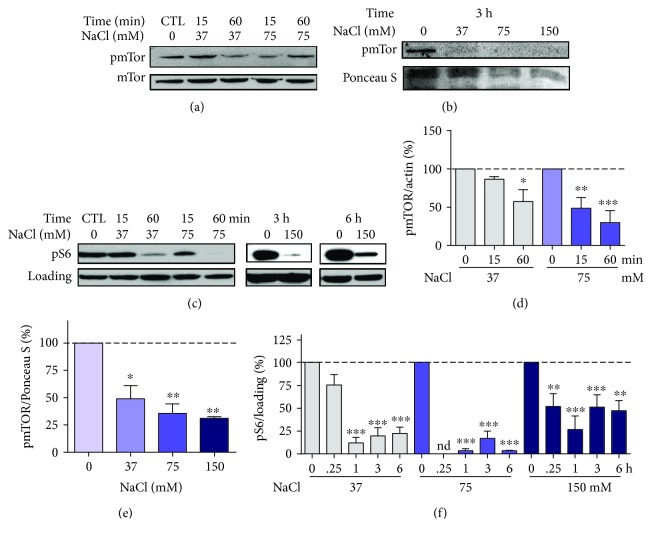
Rapid inhibition of mTOR after hyperosmotic stress in HT22 cells. Cells were treated for the indicated times and the indicated NaCl concentrations. Representative blot images of the phosphorylated (pmTOR) and total forms of mTOR at (a) 15-60 min (*N* = 3 − 4) and (b) 3 h (*N* = 2), along with their respective quantification (d and e), and S6 ribosomal protein (pS6) blot image (c) and its respective quantification (f). For pS6 ribosomal protein, *α*-AMPK (15 and 60 min) and *β*-actin (3 and 6 h) were used as the loading controls, and sample size was 3-6, except for 6 h and 75 mM, which are presented as the average of two independent experiments. nd = not determined. Values are presented as mean ± SEM. Statistical significance is presented as ^∗^*p* < 0.05, ^∗∗^*p* < 0.01, or ^∗∗∗^*p* < 0.001.

The literature reports that activation of AMPK by hyperosmotic stress is accompanied by a decrease in mTOR phosphorylation, an indication of AMPK-dependent mTOR inhibition [11]. Therefore, we evaluated mTOR phosphorylation at the same time points. Between 15 and 60 minutes after NaCl addition to the media, mTOR phosphorylation progressively decreased along with the increasing NaCl concentration (Figures 2(a) and 2(d)). The same marked decrease in mTOR phosphorylation could be observed 3 h after NaCl treatment (Figures 2(b) and 2(e)). This finding is corroborated by a parallel decrease in S6 ribosomal protein phosphorylation (Figures 2(c) and 2(f)), a known marker of mTOR activity [29]. Lower S6 phosphorylation levels were maintained for at least 6 h, even at NaCl concentrations where AMPK activation was not observed, especially within one hour after NaCl treatment.

**Figure 3:**
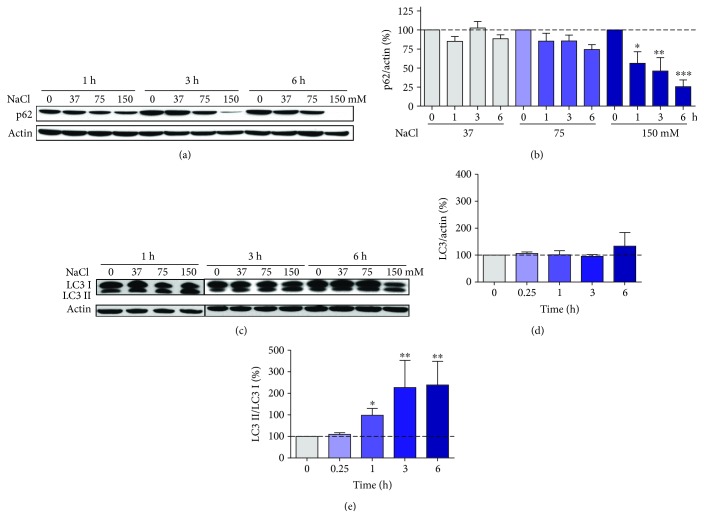
Hyperosmotic stress modulates autophagy markers LC3 and p62 in HT22 cells. Cells were treated for the indicated times and the indicted NaCl concentrations. Representative blot images of p62 (a) showing the respective quantification (b) and representative image of LC3 (c). Quantification of LC3 in cells treated with 150 mM NaCl: (d) total (LC3 I + LC3 II) and (e) the ratio of lipidated (LC3 II, lower band) to unlipidated (LC3 I, upper band) forms of LC3. Values are expressed relative to actin obtained from at least 4 independent experiments. Statistical significance is presented as ^∗^*p* < 0.05, ^∗∗^*p* < 0.01, or ^∗∗∗^*p* < 0.001.

Recent reports highlighted that downstream of AMPK activation and mTOR inhibition, hyperosmotic stress triggers autophagy [11, 13, 17]. Thus, autophagic markers were evaluated. p62 receptor protein is known to be consumed with augmented autophagy, which was also the case in HT22 cells exposed to hyperosmotic stress (Figures 3(a) and 3(b)). The highest NaCl concentration induced a time-dependent decrease in p62 levels. Six hours after induction of hyperosmotic stress, only 25% of the basal levels of p62 were observed.

Similarly, autophagy activation is usually followed by LC3 lipidation, detected as an increase in the lower molecular weight band in Western blots [30]. Hyperosmotic stress in the HT22 cells induced a time-dependent increase in the ratio of lipidated (LC3 II, lower band) to the unlipidated form (LC3 I, upper band) of LC3 (Figures 3(c) and 3(e)). The total LC3 (LC3 I + LC3 II) did not change significantly over a period of 6 h (Figure 3(d)).

**Figure 4:**
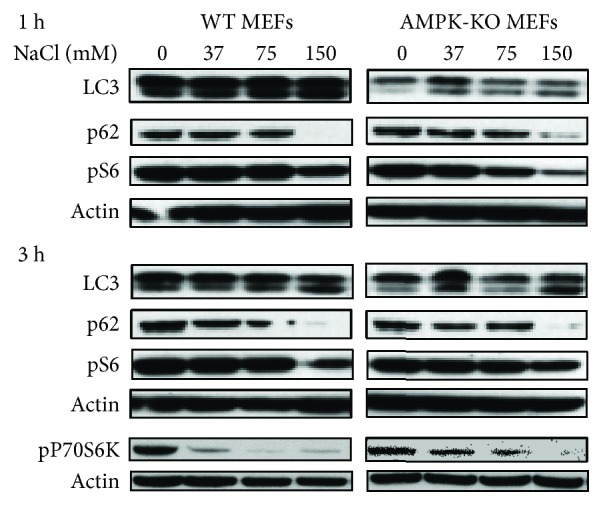
mTOR inhibition and autophagy induction were equally observed in wild-type (WT) and AMPK-deficient (AMPK-KO) mouse embryonic fibroblasts (MEFs) exposed to hyperosmotic stress. Representative images of blots probed for autophagic markers p62 and LC3 and for the mTOR pathway. Decreased p62 and an increased LC II/LC3 I ratio are markers of increased autophagy, which can be observed in both WT and AMPK-KO MEFs (compare to Figure 3(c)). The mTOR substrate, S6 kinase (P70S6K), presented lower levels of phosphorylation under hyperosmotic stress, indicating lower mTOR activity. The same can be concluded when evaluating the phosphorylated form of S6 ribosomal protein (pS6), the P70S6K substrate, which decreased at 1 and 3 hours. Images were obtained from three independent experiments.

Autophagy activation by hyperosmotic stress was reported to be AMPK-dependent [11]. Due to reports showing that cells treated with AMPK activators and inhibitors present AMPK-independent responses [31–36], we preferred to use the *α*1^−/−^/*α*2^−/−^-AMPK double mutant MEFs to test this hypothesis. Therefore, we next determined if hyperosmotic stress was able to induce autophagy in AMPK-deficient cells. First, autophagy induction by hyperosmotic stress was confirmed in WT MEFs at 1 and 3 h after hyperosmotic stress (Figure 4). Interestingly, AMPK-deficient MEFs also presented evidence for increased autophagy in response to hyperosmotic stress, as p62 was decreased and the LC3 II/LC3 I ratio was increased (Figure 4), indicating that in these cells, hyperosmotic stress-induced autophagy was independent of AMPK. Furthermore, the inhibition of the mTOR pathway in cells under hyperosmotic stress was reported to be a consequence of AMPK activation [11]. We also found indications of mTOR inhibition in WT MEFs, as expected, but, contrary to the idea that this is AMPK-dependent in response to hyperosmotic stress, we also found indications of mTOR inhibition in AMPK-deficient MEFs. This was demonstrated by the decreased phosphorylation of both P70S6K and S6 ribosomal protein, two downstream markers of mTOR activity (Figure 4).

**Figure 5:**
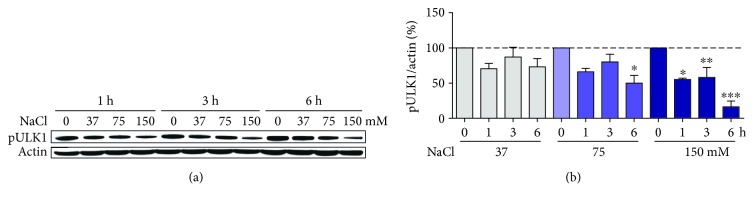
Decreased AMPK-dependent phosphorylation of ULK1 in HT22 cells under hyperosmotic stress. Cells were treated for the indicated times and indicated NaCl concentrations. Representative blot images of p^S555^-ULK1 (a) and their respective quantification (b). Values are presented as mean ± SEM of two independent experiments, except at 150 mM NaCl and 3/6 h (*N* = 4). Statistical significance is presented relative to the untreated control as ^∗^*p* < 0.05, ^∗∗^*p* < 0.01, or ^∗∗∗^*p* < 0.001.

In order to further test the idea that AMPK is not involved in autophagy induction by hyperosmotic stress, we looked at the autophagy-related AMPK substrate, serine 555 of unc-51-like kinase 1 (ULK1), an activation site. It can be clearly seen that ULK1 phosphorylation was decreased when HT22 cells were treated with NaCl (Figure 5), indicating lower ULK1 activity. This effect was most clearly observed at 150 mM NaCl, but also at 6 h and 75 mM NaCl, indicating that ULK1-dependent autophagy was decreased, rather than activated by hyperosmotic stress in these cells. This effect was observed, even at time points when AMPK was clearly activated (3-6 h after NaCl treatment) (Figure 1(c)).

### 3.2. AMPK- and Autophagy-Independent Depletion of Trx1 and Glo2

**Figure 6:**
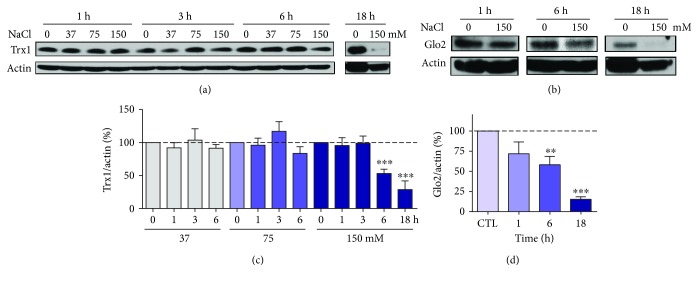
Depletion of Trx1 and Glo2 in HT22 cells under hyperosmotic stress. Cells were treated for the indicated times and with the indicated NaCl concentrations. Representative blot images of (a) Trx1 and (b) Glo2 and respective quantification relative to actin (c and d) of at least 4 independent experiments. Statistical significance is presented relative to the untreated control as ^∗∗^*p* < 0.01 or ^∗∗∗^*p* < 0.001.

In a previous study, we showed that Trx1 and Glo2 protein levels were decreased following treatment with methylglyoxal, and this loss was dependent on autophagy and AMPK [23]. Since we observed a similar increase in autophagy and AMPK phosphorylation in response to hyperosmotic stress, we decided to look at the Trx1 and Glo2 levels over time (Figure 6). As can be clearly seen, Trx1 levels were decreased at 6 and 18 h after treatment with 150 mM NaCl (Figures 6(a) and 6(c)). Likewise, this NaCl concentration produced a similar decrease in Glo2 levels (Figures 6(b) and 6(d)).

**Figure 7:**
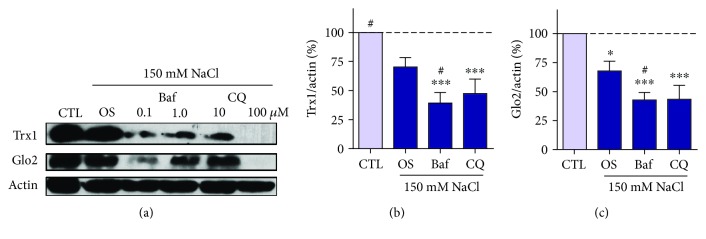
Autophagy-independent depletion of Trx1 and Glo2 in HT22 cells. The cells were pretreated for 1 h with the indicated concentrations of bafilomycin (Baf) or chloroquine (CQ) and exposed to NaCl 150 mM for 6 h (OS). (a) Representative image. Values for both concentrations of Baf (b) and CQ (c) were pooled and presented as mean ± SEM of 3 independent experiments. Statistical significance is presented relative to the untreated control as ^∗^*p* < 0.05 or ^∗∗∗^*p* < 0.001, as revealed by one-way ANOVA. An additional *t*-test analysis revealed differences indicated by ^#^*p* < 0.05, as compared to hyperosmotic stress (OS) alone.

In order to verify if Trx1 and Glo2 loss caused by hyperosmotic stress was dependent on autophagy, HT22 cells were pretreated with the late autophagy inhibitors bafilomycin (Baf) A and chloroquine (CQ) prior to hyperosmotic stress. The decrease in Trx1 (Figures 7(a) and 7(b)) and Glo2 (Figures 7(a) and 7(c)) was not abolished by autophagy inhibition with either Baf or CQ. On the contrary, autophagy inhibition seemed to facilitate Glo1 and Trx1 loss. Baf-treated HT22 cells presented lower levels of Trx1 and Glo2 as compared to hyperosmotic stress (Figures 7(b) and 7(c)). After CQ pretreatment, Trx1 and Glo2 were also decreased by hyperosmotic stress, presenting a tendency to lower levels as compared to the osmotic stress alone, but statistical differences were not significant.

**Figure 8:**
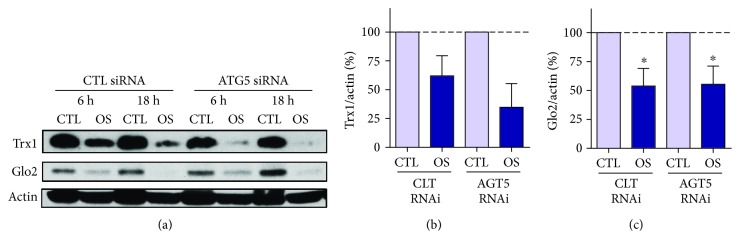
ATG5-independent depletion of Trx1 and Glo2 in HT22 cells. The cells were treated with the control or ATG5 siRNA for 48 hrs and thereafter treated with 150 mM NaCl (OS) for 6 or 18 h. Representative images (a) and respective quantification of Trx1 (b) and Glo2 (c). The effectiveness of the ATG5 siRNA in decreasing ATG5 protein has been previously reported [23]. Representative blot images of 3 independent experiments. Data from 6 h and 18 h were pooled and presented in graphs (b) and (c). Statistical significance is presented relative to the untreated control as ^∗^*p* < 0.05.

To provide further evidence that autophagy was not involved in the hyperosmotic stress-dependent loss of Trx1 (Figures 8(a) and 8(b)) and Glo2 (Figures 8(a) and 8(c)), we performed experiments with ATG5 siRNA. The response to ATG5 siRNA was similar to that observed when autophagy inhibitors were used, namely, hyperosmotic stress-induced Trx1 and Glo2 loss could not be prevented by disrupting autophagy with ATG5 siRNA.

**Figure 9:**
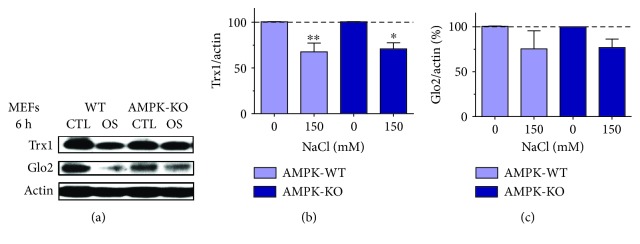
AMPK-independent depletion of Trx1 and Glo2 in wild-type and AMPK-KO mouse embryonic fibroblasts (MEFs). (a) Representative blot images of cells treated for 6 h with 150 mM NaCl (OS). Quantifications of Trx1 (b) and Glo2 (c) are presented as the average ± SEM of two independent experiments made in duplicate (*N* = 4). Statistical significance is presented relative to untreated control as ^∗^*p* < 0.05 or ^∗∗^*p* < 0.01.

Our previous findings showed that autophagic depletion of Trx1 and Glo2 in response to methylglyoxal was dependent on AMPK [23]. However, in the present work, hyperosmotic stress-dependent depletion of Trx1 and Glo2 was independent of autophagy (Figures 7 and 8). Therefore, to rule out possible AMPK participation in the depletion of Trx1 and Glo2 by hyperosmotic stress, AMPK-deficient MEF cells were treated with 150 mM NaCl for 6 h (Figure 9). Interestingly, neither Trx1 nor Glo2 hyperosmotic stress-dependent depletion could be prevented by AMPK deficiency, indicating that this event is not controlled by either autophagy or AMPK.

## 4. Discussion

### 4.1. AMPK-Independent Inhibition of mTOR and Induction of Autophagy

It has been previously shown [10–12] that hyperosmotic stress clearly induces an increase in autophagic flux, as demonstrated by increased LC3 lipidation (LC3 II), formation of LC3 puncta, p62 consumption, and transmission electron microscopy analysis. Thus, there appears to be a consensus regarding increased autophagy as a consequence of hyperosmotic stress. However, the mechanisms underlying this effect are still under debate.

Interestingly, our data showed a tendency to decreased AMPK phosphorylation shortly after hyperosmotic stress, indicating lower activity. At the same time (15-60 min), mTOR and S6 ribosomal protein showed decreased phosphorylation, which indicates that AMPK is not responsible for mTOR inhibition at this time. In line with this idea, a transient decrease in AMPK phosphorylation at phospho-T189 was also observed in yeast exposed to hyperosmotic stress [8]. We did find AMPK activation, however, at later times (3 and 6 h). Despite this activation, the essential component in canonical autophagy, ULK1 [13, 37] showed decreased AMPK target site phosphorylation (serine 555). Of note, noncanonical autophagy [38] has been demonstrated in ULK1/2 double mutant cells [39] and by ULK1/2 knockdown [40]. This further supports the notion that AMPK is not involved in the observed mTOR inhibition and autophagy activation in response to hyperosmotic stress in nerve cells.

More striking evidence that AMPK was not involved in hyperosmotic stress-induced autophagy was observed in AMPK-KO MEFs. The autophagy markers (decrease in p62 and an increase in LC3 II) were equally present in WT and AMPK-KO MEFs, ruling out a role for AMPK in hyperosmotic stress-induced autophagy. Furthermore, the mTOR pathway was clearly inhibited, as demonstrated by lower phosphorylation of P70S6K and S6 ribosomal protein, in both WT and AMPK-KO MEFs. This observation also supports the idea that AMPK is not associated with an inhibition of the mTOR pathway under these conditions.

Similar to our data, mTOR inhibition and AMPK activation were shown in HeLa and HCT116 cells exposed to hyperosmotic stress [13]. These authors showed that autophagy induction was dependent on polycystin-2, a protein involved in mechanosensation, detecting tonicity changes. The same AMPK activation and mTOR inhibition were observed in nucleus pulpous cells [11], in agreement with our findings. However, when we analyze more closely the published data, there is little evidence linking AMPK as a key factor leading to mTOR inhibition and in controlling autophagy [11, 13] in response to hyperosmotic stress. While the lower mTOR activity could be a consequence of AMPK activation [38], the authors did not test for this directly. Interestingly, hyperosmotic stress-induced autophagy in macrophages was clearly dependent on AMPK activation, which was responsible for mTOR inhibition [16]. A possible explanation is that macrophages are specialized cells and may have distinct pathways for autophagy signaling. Alternatively, these results are consistent with the idea that the mechanisms underlying autophagy induction by hyperosmotic and other stresses can be cell type-specific and need to be investigated for each cell type.

A number of literature reports highlight AMPK-independent and mTOR-independent autophagy, as previously reviewed [38]. For instance, carbonyl cyanide m-chlorophenylhydrazone (CCCP), a mitochondrial uncoupler, induced AMPK activation, although mTORC1 inhibition was independent of AMPK [41]. CCCP-induced autophagy was also independent of AMPK, which is very similar to our results with hyperosmotic stress. Similar to our results, these authors used *α*1/*α*2-AMPK double knockout MEFs. Furthermore, our results were similarly observed in HT22 cells and MEFs, suggesting that this hyperosmotic stress signaling response may have a wider distribution among cell types than previously anticipated.

### 4.2. AMPK- and Autophagy-Independent Decrease in Trx1 and Glo2

Our initial hypothesis linking hyperosmotic stress to autophagy as a potential mechanism for Trx1 and Glo2 loss, as previously shown to occur with HT22 cells treated with methylglyoxal [23], was not confirmed. The loss of Trx1 and Glo2 was not prevented by autophagy inhibition with Baf or CQ, nor with siRNA against the essential autophagy protein, ATG5. These data indicate that autophagy was not involved in the decrease in Trx1 and Glo2 seen in response to hyperosmotic stress, and therefore, this decrease must occur by a mechanism distinct from the one found for methylglyoxal. Indeed, autophagy inhibition with Baf, CQ, or siRNA against ATG5 appeared to potentiate the effects of hyperosmotic stress, as a more prominent decrease in Trx1 and Glo2 was observed. There are functional connections between the proteasome and the autophagy systems, in that the inhibition of one can lead to a compensatory upregulation of the other system [42]. A possible proteasomal degradation of Trx1 and Glo2 is an interesting point to be investigated; however, this is beyond the scope of this work.

In line with the above observations that AMPK was not responsible for autophagy induction by hyperosmotic stress, it was also not required for the decrease in Trx1 and Glo2, as this effect of hyperosmotic stress was not prevented in AMPK-KO MEFs. Further studies are required to address other potential routes for the hyperosmotic stress-dependent decrease in Trx1 and Glo2.

The endogenous toxin methylglyoxal has been closely associated with Parkinson's [43], Alzheimer's [44], vascular [45], and metabolic [46] diseases. Regarding potential consequences due to lower Glo2 levels, we anticipate that lower Glo2 protein levels in cells under hyperosmotic stress should make them more sensitive to methylglyoxal. It has been shown that lower Glo2 activity increases cellular vulnerability to methylglyoxal [47]. Furthermore, similar to the methylglyoxal effect, hyperosmotic stress was also responsible for lower Trx1 protein. Trx participates in cellular redox homeostasis and antioxidant protection [21, 48]. Peroxiredoxins are important peroxidases that depend on Trx to be regenerated in the catalytic cycle [22, 49]. Thus, lower Trx levels, in the presence of hyperosmotic stress, are expected to disturb cellular redox signaling and to impair peroxide removal.

## 5. Conclusions

Dehydration naturally occurs in humans and other animals for a number of reasons, including in the context of ageing and neurodegenerative diseases [4, 6, 7]. Of note, hyperosmotic stress clearly activates autophagy in nerve cells as previously reported in several studies using other types of cells [9–12]. The high relevance of hyperosmotic stress to nerve cells and the limited number of mechanistic studies available prompted us to investigate the role of the AMPK-mTOR pathway in HT22 nerve cells exposed to hyperosmotic stress since autophagy induction by hyperosmotic stress has been proposed to be mediated by AMPK-dependent mTOR inhibition [13, 16]. Although AMPK activation was observed, our data indicate that this was not responsible for either mTOR inhibition or autophagy induction, thus pointing to alternative mechanisms for the modulation of these pathways in HT22 cells and MEFs under hyperosmotic stress. Furthermore, the decrease in Trx1 and Glo2 is autophagy- and AMPK-independent, just the opposite from what was observed when AMPK and autophagy were induced by methylglyoxal [23]. The mechanisms responsible for both mTOR inhibition and the loss of Trx1 and Glo2 in nerve cells and MEFs exposed to hyperosmotic stress are interesting topics for future research.

## Data Availability

The data used to support the findings of this study are available from the corresponding author upon request.

## Abbreviations

AMPK: 5′AMP-activated protein kinase
Baf: Bafilomycin
CQ: Chloroquine
Glo: Glyoxalase
MEFs: Mouse embryonic fibroblasts
mTOR: Mechanistic target of rapamycin
LC3: Microtubule-associated protein 1 light chain 3
P70S6K: p70 S6 kinase
S6: Ribosomal protein S6
Trx: Thioredoxin
ULK1: Unc-51-like kinase 1.
